# Safety and Efficacy of Brachytherapy in Inoperable Endometrial Cancer

**DOI:** 10.3390/jpm14121138

**Published:** 2024-12-03

**Authors:** Ricarda Merten, Vratislav Strnad, Claudia Schweizer, Michael Lotter, Stephan Kreppner, Rainer Fietkau, Philipp Schubert, Andre Karius

**Affiliations:** 1 Department of Radiation Oncology, Universitätsklinikum Erlangen, Friedrich-Alexander-Universität Erlangen-Nürnberg, Universitätsstrasse 27, 91054 Erlangen, Germany; 2 CCC Erlangen-EMN, Comprehensive Cancer Center Erlangen-EMN (CCC ER-EMN), 91054 Erlangen, Germany; 3 CCC WERA: Comprehensive Cancer Center Alliance WERA (CCC WERA), 91054 Erlangen, Germany; 4 BZKF: Bavarian Cancer Research Center (BZKF), 91054 Erlangen, Germany

**Keywords:** inoperable, endometrial cancer, hemostyptic irradiation, brachytherapy, definitive treatment

## Abstract

**Background/Objectives**: Radiotherapy represents the only treatment option for patients with inoperable endometrial cancer (EC). The aim of our study was to evaluate the efficacy and safety of brachytherapy (BT) in this selected patient population. **Methods**: Between 1990 and 2019, 18 patients with inoperable EC in stage FIGO I–IV were treated with intracavitary brachytherapy using the “Heyman Packing technique”. BT was performed either as sole PDR- or HDR-brachytherapy with a median cumulative dose up to 60.0 Gy (67.9 Gy _EQD2 α/β = 3Gy_) and 34.0 Gy (75.6 Gy _EQD2 α/β = 3Gy_), respectively. **Results**: The median follow-up was 46 months (6–219). The mean age was 71 years. The 5-year cumulative local recurrence rate (CLRR) for the whole cohort was 27.3%. The 5-year overall survival (OS), distant metastasis-free survival (DMFS), and disease-free survival (DFS) were 51%, 79%, and 69%. The 5-year DFS for low-, intermediate-, and high-risk EC was 89%, 50%, and 44% (*p* = 0.51). No significant difference in DFS was observed in patients over 70 (*p* = 0.526). No late side effects of grade > 1 were documented. **Conclusions**: Brachytherapy for inoperable EC is a safe and effective treatment option, offering good local control and OS with minimal toxicity. Moreover, brachytherapy effectively controls hemoglobin-relevant bleeding. Therefore, BT should be considered a viable alternative to non-curative treatment strategies in gynecological multidisciplinary conferences.

## 1. Introduction

In recent years, the treatment of endometrial cancer (EC) has considerably advanced. EC has traditionally been categorized into two main types: type I endometrioid adenocarcinoma (80–90%) and type II non-endometrioid subtypes (10–20%), including serous, clear cell, and undifferentiated carcinomas [1].

Advancements in pathology and improved diagnostic methods have led to the identification of several molecular risk factors for endometrial cancer, which are essential for therapeutic decision-making and are now part of general guidelines [2].

Endometrial cancer is currently categorized into molecular subtypes: POLE-mutated, microsatellite instability (MSI), p53-aberrant, and no specific molecular profile (NSMP). For example, the POLE mutation, which is found in 7–12% of cases, is associated with a high mutation burden and a better prognosis, often allowing the omission of adjuvant therapy [3,4,5]. In contrast, aberrant p53 or expression of L1CAM benefit most from systemic or combination therapies [6,7]. In the early stages, surgical treatment comprising total abdominal hysterectomy and bilateral salpingo-oophorectomy represents the established standard of care, with postoperative radiotherapy recommended based on stage and risk factors [2,8]. Notably, the PROTEC-3 trial showed that adjuvant radiotherapy and chemotherapy significantly improve failure-free survival (*p* = 0.02) in high-risk patients [9,10].

Despite these positive developments, there remains a small cohort of patients who, due to multimorbidity, including comorbidities such as arterial hypertension, obesity, diabetes, and renal insufficiency, are neither eligible for systemic therapy nor surgery. Considering demographic changes, the number of older female patients is steadily increasing. Additionally, due to lifestyle factors, particularly in industrialized nations, there is a strong increase in obesity and comorbidities [11]. The main risk factors for developing endometrial cancer include endocrine factors such as nulliparity, obesity, diabetes, arterial hypertension, and metabolic syndrome [12,13,14]. As a result, we will increasingly face the challenge of introducing patients to standard therapy—either systemic therapy or local therapy. Due to the low incidence of inoperable cases, prospective studies on definitive radiotherapy remain scarce. However, several reviews and case reports have documented excellent outcomes with brachytherapy alone, showing long-term remissions between 60 and 80% [15,16]. Five-year overall survival rates of 42–90% can be achieved, though some data originate from the 2D era [15]. More recent studies report local control (LC) rates of over 90% and an OS of 76% at 2 years [16,17,18]. As a consequence, it is crucial that brachytherapy is discussed and well recognized as a possible alternative to hysterectomy and as an important alternative to ‘best supportive care’ for future discussions in gynecological tumor boards.

Our analysis aimed to confirm the feasibility and efficacy of intracavitary brachytherapy [19,20].

## 2. Materials and Methods

Between 1990 and 2019, eighteen patients with histologically confirmed endometrial cancer were treated with intracavitary brachytherapy instead of hysterectomy. The data analysis was conducted in 2024. Patients with FIGO stage I–IV disease were included. The risk groups were determined according to ESGO/ESTRO/ESP guidelines based on FIGO stages and clinical factors such as lymphatic vessel invasion and tumor grading. The categories are classified as low risk (FIGO IA), intermediate risk (FIGO IB), and high risk (FIGO II–IV) [21]. All patients were deemed inoperable due to obesity or significant comorbidities, including diabetes, cardiac diseases, and/or arterial hypertension. A non-hemodynamic pulmonary embolism was not considered a contraindication for brachytherapy. In general, vaginal bleeding was an indication for definitive radiotherapy. All patients received brachytherapy alone, in accordance with our clinical standard. Patient and tumor characteristics are summarized in Table 1 and Table 2.

Intracavitary brachytherapy was performed using either PDR or HDR techniques with Iridium-192 for all patients. For HDR brachytherapy, doses of 6–8, 5 Gy per fraction were administered, with a median total dose of 34.0 Gy (75.6 Gy _EQD2 α/β = 3Gy_). PDR brachytherapy was delivered continuously at 0.5–0.8 Gy per hour, with a median total dose of 60.0 Gy (67.9 Gy _EQD2 α/β = 3Gy_). Brachytherapy was performed under laryngeal mask anesthesia, markedly reducing the anesthetic risk. Given the patients’ obesity and multimorbidity, intubation anesthesia would have posed higher risks. Subsequently, initially, Norman Simon Applicators (Elekta, Nucletron) “Heyman capsules” (tube applicators with an ovoid end, diameter 4.6 or 8 mm) were inserted under general anesthesia for the majority of patients. The use of Heyman capsules enables flexible adaptation to the individual uterine anatomy. In two patients, a tandem was used instead of Heyman capsules due to unfavorable uterus anatomy. For the implantation, the uterus was visualized using transvaginal and transrectal ultrasound, and the cervix was stabilized with two uterine tenaculum forceps. The cervical canal was then dilated using a Hegar dilator up to size 8. Following maximum dilation, the “Heyman capsules” were positioned sequentially. Fixation was achieved with firm vaginal gauze packing. Finally, the correct placement of the applicators was confirmed using transrectal ultrasound, similar as for cervical cancer [22]. Throughout the treatment, the patient remained on strict bed rest in a shielded area. At the end of the treatment, the applicators were removed, and the patients were discharged from the hospital the following day. Treatment characteristics are summarized in Table 3.

Between 1990 and 2010, we specified the dose on Point My (Lit), and since 2010, CT-based clinical target volume has been performed based on diagnostic imaging findings from transvaginal ultrasound and computed tomography (CT). Endometrial thickness was used to define the high-risk clinical target volume (HR-CTV). Furthermore, we delineated the anatomically adjacent organs at risk (OARs) for the rectum and bladder for all patients. If relevant for the CTV due to anatomical positional variability, we also documented the dose values for sigmoid according to the recommendations [19,23]. To determine the total reference dose for the patients receiving combined treatment, the cumulative doses from EBRT and brachytherapy were calculated and converted to EQD2 using alpha/beta values of 10 Gy for HR-CTV and 3 Gy for OARs. The EQD2 dose (equivalent dose in 2 Gy fractions) describes the biological effectiveness of a radiation dose adjusted for fraction size. The calculations are based on the linear-quadratic model, using α/β values to represent the response of tumors (10 Gy) and normal tissues (3 Gy) to radiation. This approach facilitates the comparison of doses between different brachytherapy techniques.

Dose specification using 2D imaging in intracavitary therapy planning was conducted by 10/18 patients (55.5%). A reference point, termed ‘Point My’, was established 2 cm cranially from the applicator tip and 2 cm laterally from the long axis of the uterine cavity, with doses prescribed in accordance with ICRU 38 guidelines [24,25]. During the 2D treatment planning era, the optimal shape of isodose lines was based on the expertise of the radiation oncologist using the visualization of isodoses on available two-dimensional imaging. At that time, accurate calculation of dose–volume histograms (DVHs) was not feasible, and dose estimations were instead based on recommended reference points at organs at risk (OARs) [25,26,27,28]. The volume could only be presented for the isodoses as a cumulative, differential, or natural dose–volume histogram (DVH) value [24]. Dose rate calculations followed the TG-43 report algorithm [28].

For 8/18 patients (44.4%), the dose prescription was based on the D90 value (the dose the most exposed 90% of the target structure receives). For PDR-BT, a repair half-time of 1.5 h was additionally estimated for primary cancer tissue [29]. Furthermore, a reduced repair half-time of 0.4 h was assumed for the bladder and rectum [29,30]. The EQD2 calculation followed the incomplete LQ repair model as described previously [31].

**Figure 1:**
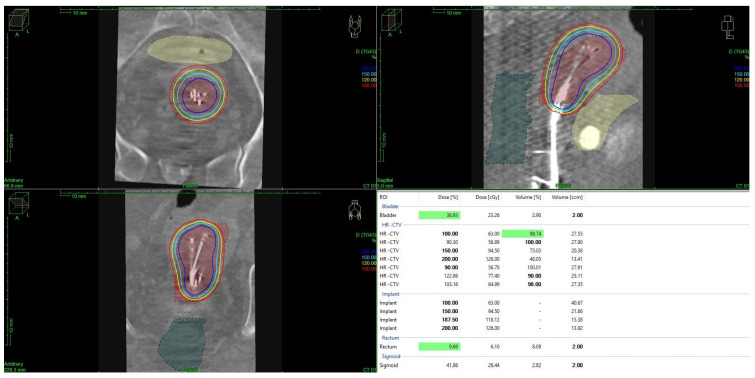
Brachytherapy using Heyman packing techniques and dose distribution in a patient with endometrial cancer. Graphic created with Oncentra Brachy (Nucletron, The Netherlands).

For both HDR- and PDR-brachytherapy, the dose constraints for organs at risk were set to a D2ccm (dose the most exposed 2ccm of the structure receives) of <75 Gy for the rectum and <85 Gy for the bladder, respectively. Dose calculation and treatment planning for brachytherapy was performed using TPS Plato and Oncentra Brachy (Nucletron, The Netherlands), with dose distributions being optimized through geometrical and graphical techniques. The treatment plan evaluation was based on dose–volume histogram (DVH) metrics, including D90, D100, V100, and V150 for the target volume and D2ccm for the OARs, as mentioned above (see Figure 1).

### Statistical Analysis

Statistical analysis was performed using SPSS v28.0 (IBM Corp, Armonk, NY, USA). The outcome was analyzed in terms of CLRR, OS, DFS, and DMFS rates at 5 years. Cumulative local recurrence rates, overall survival, distant metastasis-free survival, and disease-free survival (DFS) were defined from the last date of our treatment (determined as the date of the end of BT) until death or last contact. Reported survival rates were calculated according to Kaplan–Meier estimates.

## 3. Results

The mean age of the patients was 71 years (range: 51–90), and the median follow-up was 46 months (range: 6–219). There were 12/18 (66.7%) patients staged FIGO IA/B, 3 (16.7%) patients with FIGO II, and 2 (11.1%) patients with FIGO IV. Type I endometrial cancer was diagnosed in 15 (83.3%) patients, while 3 patients had type II. Lymphatic metastasis was present in two and hematogenic metastasis in two (11.1%) patients. The hormone receptor status for estrogen and progesterone was positive in seven (38.9%) patients. Receptor status was not documented for 11 (61.1%) patients. Eight (44.4%) of the patients also had arterial hypertension, seven (38.9%) diabetes mellitus, and six (33.3%) concomitant cardiac disease (e.g., CHD, VHF, or heart failure). Additionally, 13 patients were diagnosed with morbid obesity, with a median BMI of 42.2 (range: 21–70). Weight was not documented for four (22.2%) patients. The median number of births was two (range 0–4).

All patients received definitive radiation therapy. A total of 16 (88.9%) patients had primary endometrial cancer, while 2 (11.1%) had recurrent disease. Intracavitary brachytherapy was performed using the pulsed dose rate (PDR) technique in nine patients (50%) and the high dose rate (HDR) technique in nine patients (50%). A median of nine Heyman capsules was used during BT. Dose calculation was based on the D90 parameters in eight (44.4%) and point My in ten patients (55.6%). Dose constraints regarding organs at risk were adhered to in all patients. Further dosimetric data for brachytherapy treatments and OARs are summarized in Table 4. Two patients were not included in the dose calculation for sole HDR. They discontinued after one fraction, following successful control of tumor bleeding.

**Figure 2:**
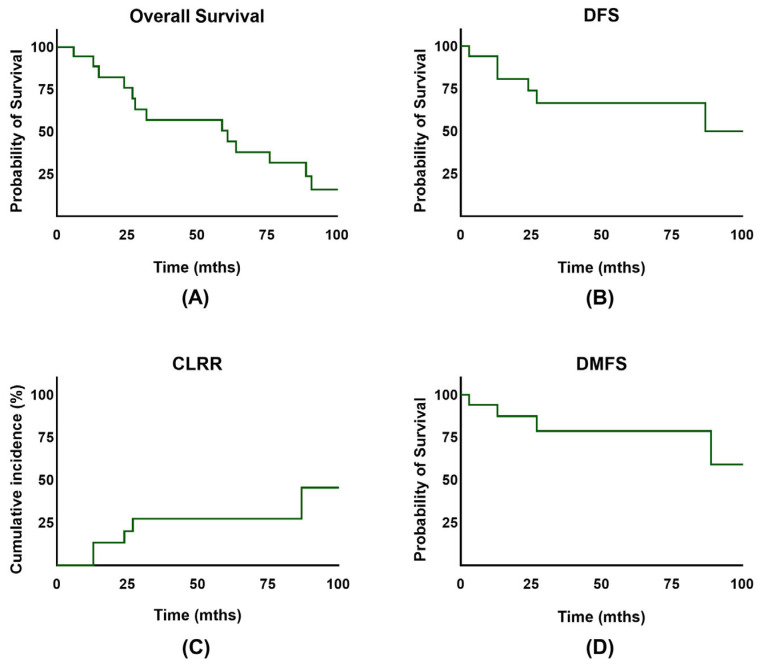
(**A**) Overall survival (OS) of patients with endometrial cancer. (**B**) Disease-free survival (DFS) of patients with endometrial cancer. (**C**) Cumulative incidence for competing event recurrence in patients after treatment. (**D**) Distant metastasis-free survival (DMFS) of patients with endometrial cancer.

**Figure 3:**
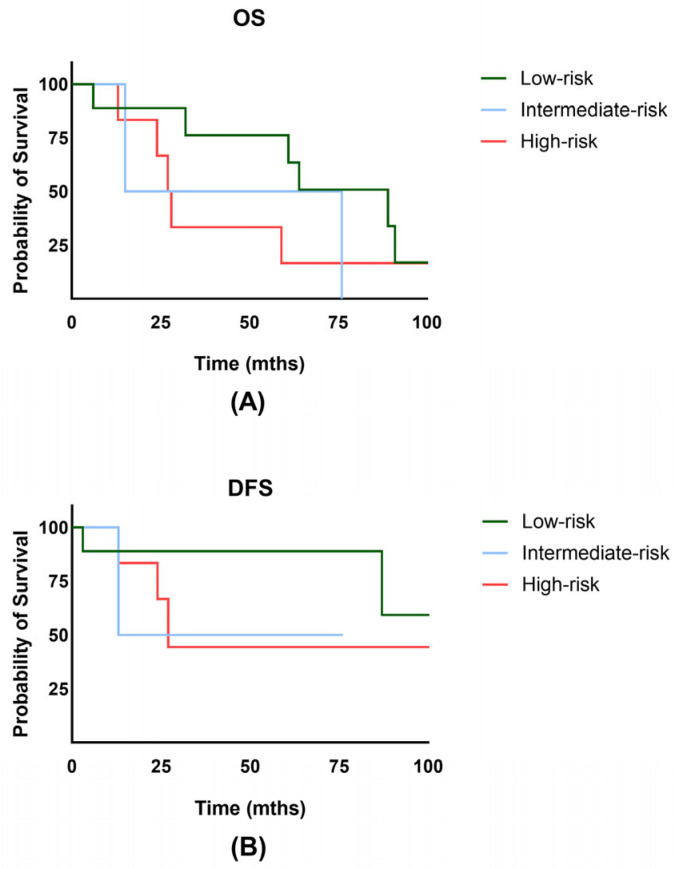
(**A**) Overall survival (OS) of patients with low-, intermediate-, and high-risk endometrial cancer. (**B**) Disease-free survival (DFS) of patients with low-, intermediate-, and high-risk endometrial cancer.

The overall survival rate for the entire cohort at 2 and 5 years was 75.9% and 50.6% (Figure 2A). Notably, the disease-free survival rate at 2 and 5 years was 75.6% and 68.7% (Figure 2B). No significant difference in OS (*p* = 0.86) or DFS (*p* = 0.24) was observed between the HDR and PDR groups. Overall, vaginal bleeding could be stopped in 15 of the patients (83.3%). There were 4/18 patients (22.2%) who experienced a local recurrence in the course of their lifetime, and 2 patients (11.1%) showed progression or persistence of the disease. Importantly, 2/18 initially advanced patients had FIGO stage IV disease. The 5-year cumulative local recurrence rate for the whole cohort was 14.9% (Figure 2C). Distant metastasis occurred in 5/18 patients (27.8%), with 2/18 patients developing lymph node recurrence and 4/18 experiencing distant/hematogenic metastasis. The 2- and 5-year DMFS rates were 87.7% and 78.9% (Figure 2D). The 5-year OS for low-risk was 76.2%, for intermediate-risk 50%, and for high-risk EC 33.3% (*p* = 0.654) (Figure 3A). The 5-year DFS for low-risk was 88.9%, for intermediate-risk 50.0%, and for high-risk EC 44.4% (*p* = 0.506) (Figure 3B). Additionally, no significant difference in disease-free survival was observed in older patients (*p* = 0.526). Further subgroup analysis for the FIGO stage was not performed due to the small number of patients in FIGO stages II and IV.

Acute and late side effects were documented based on Common Toxicity Criteria (CTC) and LENT SOMA criteria [32,33]. No acute or late adverse effects > grade 1 were observed.

## 4. Discussion

The aim of our analysis was to demonstrate the efficacy and feasibility of brachytherapy in patients with inoperable endometrial cancer. While the small sample size limits our findings, this study provides valuable insights into the use of brachytherapy in this population. It is important to note that current data indicate that only 3–9% of patients with endometrial cancer are classified as inoperable, resulting in a relatively small patient cohort that requires alternative treatment methods [15].

Doubtless, for operable endometrial cancer, surgery remains the gold standard, typically involving a total hysterectomy with bilateral salpingo-oophorectomy [8]. Early-stage patients, especially those at low and intermediate risk, benefit with 5-year survival rates of 78–90% and >75%, respectively [34,35,36,37]. However, these rates can vary considerably depending on comorbidities and age. Although minimally invasive techniques are now also standard for older patients, such procedures are often associated with a higher rate of complications. One major risk, particularly in multimorbid and obese patients, is increased aspiration. Due to the need to insufflate the abdominal cavity with CO_2_ gas to create sufficient space for instruments, intra-abdominal pressure is increased, which requires intubation anesthesia. In contrast, brachytherapy can be safely performed with laryngeal mask anesthesia, reducing perioperative risks associated with general anesthesia.

Recommendations for postoperative radiotherapy, whether vaginal brachytherapy and/or external beam radiation therapy, depends on the postoperative tumor stage and related risk factors, as it markedly reduces the risk of local recurrence [8,38,39,40]. The PORTEC-2 study showed vaginal brachytherapy was superior to pelvic radiotherapy in intermediate-risk patients, while PORTEC-3 demonstrated a 7% improvement in progression-free survival in high-risk cases receiving chemoradiotherapy but no overall survival benefit [41,42,43].

Despite all these major advances in recent decades, we are still unable to offer optimized and individualized therapy to all patients with endometrial cancer. Given the increasing prevalence of obesity and metabolic syndrome in the total population of the world, treating patients with these conditions remains challenging [13,34,35]. Comorbidities, such as obesity, diabetes mellitus, cardiac stress, and/or renal insufficiency, often make surgery or definitive chemoradiation not even feasible.

For these patients, brachytherapy (±external beam radiation therapy) is often the only treatment option. While today’s inoperable patients are typically treated with a combination of external beam radiation therapy and chemotherapy, it is important to recognize that during our treatment period, EBRT and chemotherapy were associated with significantly higher rates of side effects compared to current standards. Given the poor general health of many patients, brachytherapy alone was often the most appropriate choice. Additionally, the weight capacity of radiotherapy treatment couches was lower at the time. The precision of patient positioning on linear accelerators was also less accurate, and advanced control mechanisms such as cone-beam imaging were not as fully developed as they are today. Most of our patients had early-stage (low-risk) tumors, for which, even today, EBRT would require larger safety margins to account for uterine movement. Brachytherapy, by contrast, allows for high doses to be delivered directly to the mucosal surface, which cannot be replicated by EBRT, even with modern techniques. Brachytherapy also provides superior hemostatic effects on the endometrium through dose escalation to the mucosal surface, making it particularly beneficial for this patient group with active bleeding.

It has to be noted that due to the varying treatment regimens (brachytherapy alone, EBRT alone, or combination therapy) and treatment planning methods (2D vs. 3D planning), survival rates differ widely [19,44]. Furthermore, the availability of advanced diagnostics, specialized centers, and access to linear accelerators and brachytherapy applicators varies across healthcare systems. The key question remains as to whether the benefits truly outweigh the potential side effects.

Despite increasing recognition of endometrial cancer and the growing number of older patients with comorbidities, the literature on inoperable endometrial cancer remains limited, often consisting of small and outdated case studies [15,16,18,45,46,47]. Even with a reduced life expectancy, definitive brachytherapy in medically inoperable, multimorbid patients provides effective local tumor control and sustained hemostasis. Arians et al. demonstrated local control of 86% in stage IA, 68% in stage IB, and 60% in stage II, with 5-year disease-specific survival rates of 84%, 73%, and 68%, respectively [16]. An additional evaluation of a similarly small patient group reported local control rates of 86% at 3 years and 69% at 6 years [48]. These results are in line with our data. Gebhardt and Gannavarapu et al. were also able to demonstrate good local control, cancer-specific survival, and overall survival in their analysis, even with a smaller sample size [18,49]. In low-risk patients, Gebhardt showed excellent locoregional control rates of 90% and cancer-specific survival rates of 97% [49]. However, the median follow-up was only 17 and 18 months [18,49]. This shows the need for close follow-up and possibly the inclusion of adjuvant therapies in high-risk patients as far as possible.

The analysis of our patient cohort treated for inoperable endometrial cancer using a “Heyman packing technique” provides encouraging results, particularly given the limited treatment options available for this patient group. Nevertheless, several critical aspects still need to be discussed.

We recognize that comparisons with standard therapy are difficult due to our small sample size. However, we believe that our results have considerable value for this vulnerable subgroup of patients with limited treatment options.

The mean age of the patients was 71 years, reflecting the older demographic typically affected by this disease. The high prevalence of comorbidities, such as arterial hypertension (44%), diabetes mellitus (39%), and concomitant cardiac disease (33%), is notable. These comorbidities not only affect the patient’s quality of life but also their treatment tolerance and overall prognosis. Additionally, the high incidence of morbid obesity, with a median BMI of 42.2, likely influenced both the choice of treatment and the risk of side effects.

An important finding is the incomplete assessment of hormone receptor status, which was missing in 61% of the patients due to the earlier treatment periods of some patients. This represents an essential limitation, as hormone receptor status is known to impact prognosis and treatment options. Still, palliative hormonal therapy needs to be seen as critical in vulnerable patient groups with severe comorbidities due to the increased risk of thromboembolic events. Despite these weaknesses, our clinical results are promising. Vaginal bleeding stopped in 83% of patients, and the recurrence rate was 33.3%. It is important to acknowledge that the accuracy of tumor classification may have been affected by the long observation period, which dates back to the early 1990s. In the early years of this study, imaging and classification techniques were less advanced, potentially leading to the misclassification of tumors. This could explain the local recurrence rate. Today, advances in imaging quality and more precise classification methods would probably lead to a more accurate risk assessment. These outcomes are particularly relevant, considering that surgery was not an option for these patients. The DFS rate of 68.7% at 5 years is very positive, given the lack of treatment options. It demonstrates the efficacy of this treatment approach in a situation where treatment options are limited. It should also be mentioned that 2D planning was used for the majority of our patients (60%) due to the long evaluation period. It is important to note that treatment planning modalities have significantly advanced over the extended observation period. In recent decades, brachytherapy has particularly benefited from improvements in imaging technology. Initially, brachytherapy was planned in two dimensions using orthogonal X-ray images with point dosimetry. Without defined contours for organs at risk (OARs) or target volumes, accurate dose–volume histogram (DVH) calculations were not feasible, and dose estimations relied on recommended reference points, resulting in less precision compared to modern CT- or MR-based planning. Today, image-guided brachytherapy has become a crucial part of brachytherapy workflows, allowing for far greater accuracy in treatment. [50]. Dankulchai concludes in his review that local control rates are better with 3D brachytherapy compared to 2D brachytherapy [51]. Despite good local control, the incidence of distant metastases (22.2%) remains a concern, indicating the aggressiveness of the disease in certain patient groups. In addition, we were able to show that brachytherapy is a well-tolerated treatment option for this vulnerable patient group with low acute and late toxicity (≤grade 1).

Of course, the limitations of our study should be considered. The small sample size restricts the generalizability of the results and limits the potential for subgroup analyses. Additionally, the retrospective design and the absence of a randomized control group are important factors that must be acknowledged. Given the low incidence, gathering prospective data on primary radiotherapy will remain challenging. Furthermore, brachytherapy for inoperable endometrial cancer is a highly specialized treatment, likely confined to high-volume tertiary referral centers with extensive expertise in this area. The limited availability of sufficient case volumes and training opportunities are major constraints [52,53].

## 5. Conclusions

In conclusion, our analysis demonstrates the role of brachytherapy as a valuable treatment option for patients with inoperable endometrial cancer, particularly in cases where surgery or chemotherapy is not possible. Despite the limitations of our study, such as the small sample size and retrospective design, our results provide encouraging evidence of the efficacy of brachytherapy in achieving good local control and improving quality of life with minimal toxicity in this vulnerable patient group. The high incidence of absence of vaginal bleeding and a disease-free survival rate of 69% at 5 years support the efficacy of this treatment, particularly in patients with significant comorbidities who are otherwise difficult to treat. Moreover, brachytherapy can improve the patient’s quality of life in this selected cohort by preventing repeated hospitalizations and transfusions due to bleeding. Based on our results, it is essential that each case is critically evaluated by a tumor board before any potential treatment options are ruled out.

## Figures and Tables

**Table 1:** Patient characteristics, n = 18 (%).

Age	Median 71 (51–90)
<70	9 (50)
>70+	9 (50)
Chronic disease n (%)	18 (100)
Art. hypertension	8 (44.4)
Diabetes mellitus	7 (38.9)
Adipositas per magna	13 (72.2)
Pulmonary embolism	3 (16.7)
Concomitant cardiac diseases	6 (33.3)
COPD	2 (11.1)
Renal dysfunction	5 (27.8)
Parity	Median 2 (0–4)
Weight (kg)	112.2 (58–171)
BMI	42.2 (21–70)
Hb (g/dL)	11.5 (8.2–15.3)

Abbreviations: n, number of patients; kg, kilogram; BMI, body mass index; COPD, chronic obstructive pulmonary disease.

**Table 2:** Tumor characteristics, n (%).

FIGO Stage	
I	7 (38.9)
IA	2 (11.1)
IB	3 (16.7)
II	4 (22.2)
IV	1 (5.6)
IVB	1 (5.6)
TNM-T Stage	
T1	12 (66.7)
T2	4 (22.2)
T4	1 (5.6)
unknown	1 (5.6)
TNM-N Stage	
0	14 (77.8)
1	2 (11.1)
unknown	2 (11.1)
TNM-M Stage	
0	14 (77.8)
1	2 (11.1)
unknown	2 (11.1)
Lymphinvasion	
0	3 (16.7)
1	1 (5.6)
unknown	14 (77.8)
Grading	
G1	5 (27.8)
G2	11 (61.1)
G3	1 (5.6)
unknown	1 (5.6)
Histologic subtypes	
Type I	15 (83.3)
Type II	3 (15)
Risk group	
Low	9 (50.0)
Intermediate	3 (16.7)
High	6 (33.3)
Hormone receptor status	
Estrogen receptor-positive	7 (38.9)
Progesterone receptor-positive	7 (38.9)
unknown	11 (61.1)

Abbreviations: FIGO, International Federation of Gynecology and Obstetrics.

**Table 3:** Treatment characteristics, n (%).

BT Technique	
HDR	9 (50)
PDR	9 (50)
Dose specification	
D90	8 (44.4)
My	10 (55.6)
Median numbers of applicators	9 (3–17)

Abbreviations: PDR, pulse dose rate; HDR, high dose rate.

**Table 4:** Detailed dosimetric data (interquartile ranges).

Characteristics (Gy)	Median Fraction Dose	Median Cumulative Dose	EQD2 (α/β = 3 Gy)
Sole brachytherapy			
HDR technique (n = 7)			
HR-CTV	8.0 (6.0–8.5)	34.0 (24.0–42.0)	75.6 (43.2–84.0)
PDR technique (n = 9)			
HR-CTV	0.6 (0.5–0.8)	60.0 (49.8–72.7)	67.9 (61.9–105.5)
Organs at risk			
D_2ccm_ rectum		19.6 (5.8–36.4)	12.9 (3.6–25.3)
D_2ccm_ bladder		41.9 (22.1–63.9)	30.0 (14.5–48.9)

Abbreviations: PDR, pulsed dose rate; HDR, high dose rate; Gy, gray; D_2cc_ = minimum dose delivered to most exposed 2ccm; HR-CTV, high-risk clinical target volume.

## Data Availability

The datasets generated during and/or analyzed during the current study are not publicly available due to data privacy restrictions but are available from the corresponding author on reasonable request.

## Abbreviations

AF	Atrial Fibrillation
BMI	Body mass index
BT	Brachytherapy
CHD	Coronary Heart Disease
CLRR	Cumulative local recurrence rate
COPD	Chronic obstructive pulmonary disease
CT	Computed tomography
CTC	Common Toxicity Criteria
CTV	Clinical target volume
D2ccm	Minimum dose delivered to most exposed 2ccm
3DCRT	Three-Dimensional Conformal Radiation Therapy
DFS	Disease-free survival
DMFS	Distant metastasis-free survival
DVH	Dose–volume histogram
EBRT	External beam radiation therapy
EC	Endometrial cancer
FIGO	International Federation of Gynecology and Obstetrics
Gy	Gray
HDR	High dose rate
HR CTV	High-risk clinical target volume
LC	Local control
MR	Magnetic Resonance Tomography
OAR	Organ at risk
OS	Overall survival
PDR	Pulsed dose rate
